# Potential impact of metabolic syndrome on cognitive function in US firefighters

**DOI:** 10.3389/fpubh.2023.1150121

**Published:** 2023-05-25

**Authors:** Myong-Won Seo, Joshua Gann, Jung-Min Lee, Kevin S. Heffernan, Joon Young Kim, Hyun Chul Jung

**Affiliations:** ^1^Department of Exercise Science, David B. Falk College of Sports and Human Dynamics, Syracuse University, Syracuse, NY, United States; ^2^Department of Kinesiology, School of Allied Health, University of Louisiana at Monroe, Monroe, LA, United States; ^3^Sports Science Research Center, Kyung Hee University, Yongin-si, Republic of Korea; ^4^Department of Physical Education, College of Physical Education, Kyung Hee University, Yongin-si, Republic of Korea; ^5^Department of Sports Coaching, College of Physical Education, Kyung Hee University, Yongin-si, Republic of Korea

**Keywords:** firefighter health, occupational risk, cardiometabolic disease risk, cognitive health, line-of-duty deaths

## Abstract

**Objectives:**

Among US firefighters, sudden cardiac arrest and psychological stress (i.e., PTSD) are the leading cause of on-duty death. Metabolic syndrome (MetSyn) may influence both cardiometabolic and cognitive health. Here, we examined differences in cardiometabolic disease risk factors, cognitive function, and physical fitness in US firefighters with vs. without MetSyn.

**Materials and methods:**

One hundred fourteen male firefighters, aged 20 to 60 years, participated in the study. US firefighters with MetSyn vs. non-MetSyn were divided by AHA/NHLBI criteria. Of them, we performed a paired-match analysis with respect to the age and BMI of firefighters with (*n* = 18) vs. without MetSyn (*n* = 18). The cardiometabolic disease risk factors included blood pressure, fasting glucose, blood lipid profiles [HDL-C, triglyceride (TG)], and surrogate markers of insulin resistance [TG/HDL-C, TG glucose index (TyG)]. The cognitive test included a psychomotor vigilance task as a measure of reaction time and a delayed-match-to-sample task (DMS) as a measure of memory, using the computer-based Psychological Experiment Building Language Version 2.0 program. The differences between MetSyn and non-MetSyn groups in US firefighters were analyzed using an independent *t*-test adjusted for age and BMI. In addition, Spearman correlation and stepwise multiple regression were conducted.

**Results:**

US firefighters with MetSyn exhibited severe insulin resistance estimated by TG/HDL-C and TyG (Cohen’s *d* > 0.8, all *p* < 0.01) compared with their age- and BMI-matched counterparts without MetSyn. In addition, US firefighters with MetSyn exhibited higher DMS total time and reaction time compared with non-MetSyn (Cohen’s *d* > 0.8, all *p* < 0.01). In stepwise linear regression, HDL-C predicted DMS total time (β = − 0.440, R^2^ = 0.194, *p* < 0.05), and TyG (β = 0.432, R^2^ = 0.186, *p* < 0.05) predicted DMS reaction time.

**Conclusion:**

US firefighters with vs. without MetSyn were predisposed to metabolic risk factors, surrogate markers of insulin resistance, and cognitive function, even when matched for age and BMI, and there was a negative association between metabolic characteristics and cognitive function in US firefighters. The findings of this study suggest that the prevention of MetSyn may be beneficial to supporting firefighters’ safety and occupational performance.

## 1. Introduction

The firefighting occupation exposes firefighters to abnormally high physical demands and dangerous environments, thus increasing the risk of injury and stress-related diseases in firefighters (1). The National Fire Protection Association (NFPA) reported that overexertion/stress/medical problems and sudden cardiac death accounted for 54% and 46% of firefighter fatalities in 2020, respectively (2). Given the graveness of the risk of injury and fatalities in the firefighter profession, comprehensive risk management is important for firefighters’ safety and health.

Firefighters endure work-related hazards such as physical exertion, trauma, toxic fumes, and sleep disorder, all of which may lead to cardiometabolic diseases (3). Metabolic syndrome (MetSyn) is a cluster of cardiometabolic disease risk factors, including abdominal obesity, dyslipidemia, hypertension, and impaired fasting glucose, contributing to the heightened morbidity and mortality risk (4). Recently, the meta-analysis reported that the global prevalence of MetSyn ranged from 12.5% (95% CI; 10.2–15.0) to 31.4% (95% CI; 29.8–33.0) (5). Especially, the prevalence of MetSyn among US adults increased from 25.3% in 1988–1994 to 34.2% in 2007–2012 (6) and from 32.5% (95% CI; 29.0–36.2) in 2011–2012 to 36.9% in 2015–2016 (95% CI; 33.9–39.9) (7). A longitudinal study demonstrated that individuals with MetSyn had a higher likelihood of suffering cardiovascular events (8). Several different criteria of MetSyn were previously suggested as risk factors for on-duty sudden cardiac events and/or death in US firefighters (9, 10). Therefore, the identification of appropriate MetSyn prevention and treatment strategies for US firefighters is clearly needed.

Firefighters need to make fast and accurate decisions in emergencies, including extinguishing fires and searching for and rescuing victims while wearing heavy and uncomfortable personal protective equipment (11). Cognitive function is the ability to have a variety of mental capacities, such as decision-making and problem-solving. Moreover, cognitive function is an important factor that could play a role in choosing coping behaviors in a hazardous fire situation. Therefore, firefighters need a higher cognitive function in order to perform their tasks safely and successfully. However, while firefighters are on-duty, firefighting job-related tasks can negatively affect cognitive function (12).

Previous studies suggest that firefighting tasks require a higher level of health-related physical fitness, such as healthy body composition (i.e., low body fat), muscle strength, muscle endurance, flexibility, and cardiorespiratory fitness (CRF) (13, 14). Furthermore, inadequate levels of physical fitness may lead to a sudden cardiac event through intense energy demand during firefighter’s job-related tasks (15). Nevertheless, Storer et al. reported that Southern California firefighters did not reach the NFPA minimum recommendations for physical fitness standards (16). Given that two-thirds of US firefighters are overweight/obese (17), above the average for the US general population (18), it can be postulated that there should be an important relationship between physical fitness, obesity-related comorbidities, and cognitive function.

The prevalence of MetSyn may be particularly high in US firefighters. This is important to underscore as MetSyn has been shown to have a negative effect on cognitive function, including processing speed, visuo-spatial abilities, and executive functioning, in the general population (19). Optimal cognitive function is necessary for occupational success and safety in fire services. Therefore, the study aimed to compare differences in cardiometabolic disease risk factors, cognitive function, and physical fitness among US firefighters with and without MetSyn. It was hypothesized that US firefighters with MetSyn would have a worse cognitive function and physical fitness compared with those without MetSyn.

## 2. Materials and methods

### 2.1. Experimental approach to the problem

A cross-sectional design was used to compare the cardiometabolic disease risk factors, cognitive function, and a battery of physical fitness in US firefighters with vs. without MetSyn. In addition, from total cohort data, two equivalent groups of firefighters with MetSyn vs. without MetSyn were selected using a rigorous paired-match with respect to age and BMI. MetSyn was defined using the American Heart Association and the National Heart, Lung, and Blood Institute (AHA/NHLBI) (20) and as having three of the following five criteria: (1) waist circumference ≥ 102 cm, (2) HDL-C < 40 mg/dL, (3) triglyceride (TG) ≥ 150 mg/dL, (4) fasting glucose ≥ 100 mg/dL, (5) systolic blood pressure (SBP) ≥ 130 mmHg, or diastolic blood pressure (DBP) ≥ 85 mmHg.

### 2.2. Participants

A convenience sampling method was performed in the study. Initially, 133 male firefighters have recruited from a mid-sized, West South-Central city, in the United States. Participants were included in the study if they were able to participate in physical activity such as physical fitness tests. If any participants did not want to participate in the study or were not able to participate in the physical fitness tests due to medical concerns, then they were excluded from the study. Prior to the experiment, all participants received an oral explanation of the study procedure, along with an explanation of the risks and benefits of study participation. Participants then completed the written consent form. Nineteen firefighters dropped out during measurement sessions due to personal reasons and scheduling conflicts. Thus, 114 participants completed this study. The study was approved by the University’s Institutional Review Board (#958-2019).

### 2.3. Procedures

Participants were instructed not to participate in vigorous physical activity at least 48 h before the study and were asked to fast 1 day before the test after 9:00 p.m. Participants visited the human performance lab at 8 a.m. and completed the following: (1) Written consent form and PAR-Q (Physical activity readiness questionnaire), (2) resting blood pressure, (3) fasting glucose and blood lipid profiles, (4) computer-based cognitive function tests, and (5) physical fitness tests.

### 2.4. Anthropometric characteristics and body composition

Participants’ body height and weight were measured using a stadiometer and a standard weight beam to the nearest 0.1 cm and 0.1 kg, respectively. Body mass index (BMI) was calculated by dividing body weight (kg) by the square of body height (m^2^). Waist and hip circumferences were measured with a measuring tape, and the waist-to-hip ratio (WHR) was calculated. The skinfold thickness method was applied to examine the body composition. Three body sites, including the upper chest, abdominal, and mid-thigh, were measured with a Lange skinfold caliper (Cambridge Scientific, Cambridge, Maryland, United States). Each site was measured two times by a trained technician, and the average score was reported in the study. The result was then used to estimate body fat percentage (21).

### 2.5. Cardiometabolic disease risk factors

Cardiometabolic disease risks factors, such as resting blood pressure, fasting glucose, and blood lipid profiles, were measured in the morning from 8 a.m. to 9 a.m. Participants were asked to perform overnight fasting. Resting blood pressure was measured with an automatic blood pressure monitor (Advantage 6021 N, American Diagnostic Corporation, New York, United States). Participants were seated in the back support chair for at least 10 min with both feet flat on the floor. The blood pressure cuff was placed on the upper arm about 2.5 cm above the antecubital space to ensure the cuff covered the brachial artery. The blood pressure was measured two times, and the average scores of SBP, DBP, and mean blood pressure (MBP) were recorded in the study. Fasting glucose and lipid profiles were measured with a portable blood analyzer (CaridoCheck Plus, PTS Diagnostics, IN, United States). First, a technician wiped the participant’s finger with an alcohol pad and dried it out. A disposable Lancet BD puncture was used to prick the small fingertip. The first drop of blood was wiped with a gauze pad, then the following drops of blood were collected with PTS Collect capillary tube and were used to analyze fasting glucose and blood lipid profiles. The level of fasting glucose, HDL-C, and TG was reported in the study. In addition, the surrogate estimates of insulin resistance, including TG/HDL-C and TyG, were used in the present study.

### 2.6. Cognitive function test

Participants underwent cognitive function tests in a quiet room, free of distractions, using the computer-based Psychological Experiment Building Language (PEBL) Version 2.0 program (22). Participants completed two different tests, including a psychomotor vigilance task (PVT) and a delayed-match-to-sample task (DMS). PVT is a simple reaction time test where the participant clicks the spacebar as quickly as possible when the red circle appears on the screen. The red circle appeared unexpectedly and ranged from 2 to 12 s between trials. DMS is a short-term memory test. First, a 4 × 4 matrix patterns filled with yellow and red squares is shown on the monitor. After a certain amount of time, the matrix pattern disappears, and then two matrix patterns appear on the screen. The participant selects the matrix pattern that was shown previously as accurately and quickly as possible. Following a few familiarization trials, participants performed 40 trials of the PVT test for 5 min to evaluate the average reaction time and performed 20 trials of the DMS test for 20 min to evaluate memory function (23). PVT correctness and reaction time, DMS correctness, total time, and reaction time were recorded in the study.

### 2.7. A battery of physical fitness tests

A battery of physical fitness tests included sit and reach, hand grip strength, vertical jump, chair sit-stand, and YMCA submaximal cycling tests. Participants performed warm-up exercises such as dynamic stretching and cycling for 5 min prior to the tests. A sit-and-reach test was used to examine flexibility. Participants were seated with legs extended, feet flat against a measurement box and placed their hands on the box, and pushed the measurement bar forward as far as possible. Participants performed three trials, and the best score was recorded in the study. Hand-grip strength test was assessed with a dynamometer (Jamar, Bolingbrook, IL, United States) and used to examine muscular strength. The dominant hand was measured with the arm extended. Participants maximally squeezed the dynamometer for approximately 5 s. Participants performed three trials, and the highest score was recorded. A vertical jump test was performed to examine the muscular power of a Just Jump System (Fitness Technology, Adelaide, Australia). Participants stood on the jump mat (27 inches × 27 inches) that is connected to a portable computer unit. The jump height is calculated based on the flying time taken as the time from the participants’ feet leaving the mat to once again contacting the mat. Participants performed three trials, and the best score was reported in the study. A chair sit-stand for 30 s was applied to examine muscular endurance. Participants performed as many full stands as possible after the “go” signal, and the number of sit and stands was recorded in the study. A submaximal YMCA cycling test was applied to estimate CRF. Participants first performed 3 min of warm-up cycling exercise with no resistance and 50 RPM, then pedaled for 3 min at 0.5 kg and recorded heart rate at 2 and 3 min in the first stage to find a steady state heart rate (<5 beats/min). Based on the heart rate response, different resistance was applied in the next 2nd and 3rd stages. Once the two consecutive 3-min cycling exercises were completed where the heart rate (HR) response was between 110 bpm and 85% age-predicted HR_max_, the test was terminated. The VO_2max_ value was calculated with the formula (VO_2max_ [mL/kg/min] = (work rate [kgm/min] × 1.8 mL O_2_/kgm) ÷ body weight (kg) + 7 mL/kg/min) (24).

### 2.8. Statistical analyses

The data were analyzed using SPSS for Windows, version 26 (SPSS Inc., Chicago, IL, United States). All data were expressed as mean (M) and strand error of the mean (SEM). The differences between MetSyn and non-MetSyn groups were analyzed using an independent *t*-test adjusted for age and BMI. Of them, 18 firefighters were selected from each group to perform a paired-match analysis with respect to age (±2 years) and BMI (±2 kg/m^2^). Correlation for Spearman’s correlation and regression for stepwise multiple linear regression was used to determine the association between cognitive function and cardiometabolic disease risk factors in US firefighters. Effect sizes were calculated using independent and dependent Cohen’s d. The statistical significance level was set at 0.05.

## 3. Results

The anthropometric characteristics, cardiometabolic disease risk factors, cognitive function, and physical fitness of the US firefighters are summarized in Table 1. Of the US firefighter at baseline, 66 participants (57.9%) had MetSyn. MetSyn group had significantly higher age, BMI, height, weight, percent body fat, waist and hip circumference, and WHR compared with the non-MetSyn group. In addition, the MetSyn group had significantly worse all cardiometabolic disease risk factors compared with non-MetSyn groups. Although no significant differences between groups were noted in cognitive function, the CRF was lower in the MetSyn group compared with the non-MetSyn group.

**Table 1:** Anthropometric, cardiometabolic, cognitive function, and physical fitness of the metabolic syndrome status in US firefighters.

		Non-MetSyn (*N* = 48)	MetSyn (*N* = 66)	*Adjusted p*-value	Cohen’s *d*
**Physical characteristics**					
	Age (year)	34.2 ± 1.3	43.1 ± 3.2	0.001^*^	0.40
	BMI (kg/m^2^)	28.3 ± 0.5	32.8 ± 0.7	0.001^*^	0.20
	Height (cm)	175.4 ± 1.0	178.2 ± 0.9	0.018^*^	0.22
	Weight (kg)	87.3 ± 1.6	104.5 ± 2.7	0.001^*^	0.31
	Percent body fat (%)	17.7 ± 0.6	23.3 ± 0.9	0.001	0.29
	Waist circumference (cm)	89.6 ± 1.0	106.2 ± 2.6	0.001	0.51
	Hip circumference (cm)	103.6 ± 0.8	108.7 ± 2.1	0.001	0.22
	WHR (ratio)	0.9 ± 0.0	1.0 ± 0.5	0.030	0.45
**Cardiometabolic risk factors**					
	SBP (mmHg)	129.9 ± 1.4	138.7 ± 1.9	0.001	0.30
	DBP (mmHg)	79.1 ± 1.1	88.4 ± 1.4	0.001	0.30
	MBP (mmHg)	99.4 ± 1.1	108.5 ± 1.5	0.001	0.34
	HDL-C (mg/dL)	59.8 ± 2.2	46.5 ± 1.7	0.001	0.39
	TG (mg/dL)	93.8 ± 5.9	117.5 ± 12.5	0.001	0.93
	Fasting glucose (mg/dL)	100.6 ± 1.2	109.5 ± 2.1	0.001	0.71
	TG/HDL-C	1.7 ± 0.1	4.1 ± 0.3	0.001	0.76
	TyG	8.4 ± 0.1	9.0 ± 0.1	0.001	1.18
**Cognitive function**					
	PVT correctness (score)	37.8 ± 0.7	38.2 ± 0.3	0.649	0.04
	PVT reaction time (msec)	321.8 ± 3.1	321.9 ± 4.0	0.980	0.15
	DMS correct (score)	26.8 ± 0.4	27.2 ± 0.4	0.538	0.30
	DMS total time (msec)	5900.8 ± 298.3	6879.5 ± 412.1	0.052	0.96
	DMS reaction time (msec)	2756.7 ± 134.7	3072.3 ± 191.8	0.168	0.91
**Physical fitness**					
	Sit and reach (cm)	24.5 ± 1.1	21.5 ± 1.2	0.065	0.71
	Hand grip strength (kg)	49.8 ± 1.1	50.7 ± 1.4	0.625	0.19
	Vertical Jump (cm)	19.3 ± 1.4	18.4 ± 1.0	0.521	0.16
	Chair sit-stand (*n*)	46.3 ± 1.4	42.3 ± 1.9	0.113	0.35
	VO_2max_ (mL/kg/min)	36.0 ± 1.6	31.1 ± 1.5	0.031	0.94

*Unadjusted *P*-value; Adjusted for age and BMI; MetSyn, metabolic syndrome; WHR, waist-to-hip ratio; BP, blood pressure; SBP, systolic BP; DBP, diastolic BP; MBP, mean BP; TG, Triglyceride; TyG, TG/glucose; PVT, psychomotor vigilance task; DMS, delayed-match-to-sample task.

**Figure 1:**
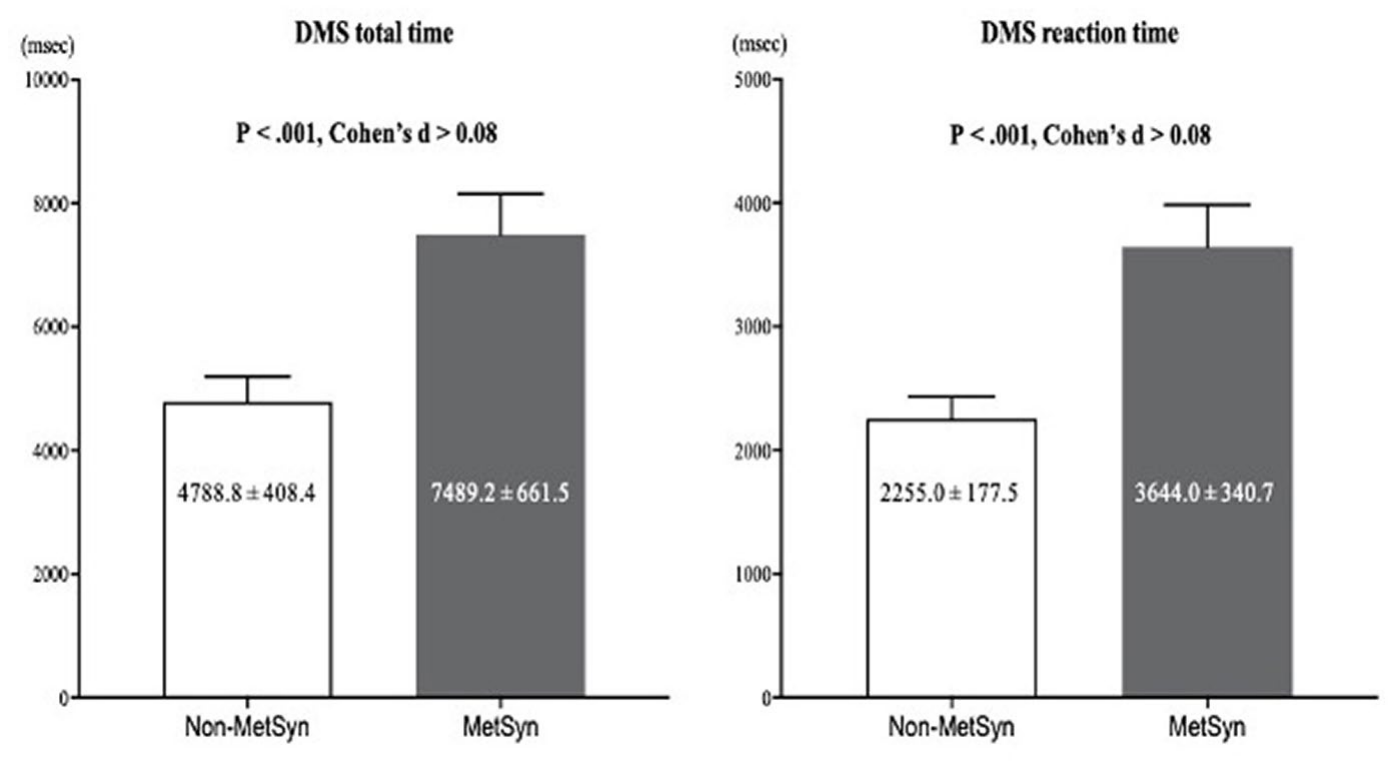
Delayed-match-to-sample task (DMS) of the paired-matched US firefighters with vs. without MetSyn. Data are mean ± SEM, MetSyn; metabolic syndrome.

For subgroup analyses (Table 2), although anthropometric characteristics and blood pressure variables were not significantly different between groups, fasting glucose, blood lipid profiles, and surrogate estimates of insulin resistance were similar to that of the total population analyses. In addition, DMS total time and DMS reaction time were higher in the MetSyn group compared with the non-MetSyn group (Figure 1), and CRF was significantly lower in the MetSyn group than in the non-MetSyn group.

**Table 2:** Anthropometric, cardiometabolic, cognitive function, and physical fitness of the paired-matched metabolic syndrome status in US firefighters.

		Non-MetSyn (*N* = 18)	MetSyn (*N* = 18)	*P*-value	Cohen’s *d*
**Physical characteristics**					
	Age (year)	40.3 ± 2.3	40.3 ± 2.6	1.000	0.00
	BMI (kg/m^2^)	29.5 ± 0.9	30.3 ± 0.6	0.433	0.20
	Height (cm)	175.3 ± 1.4	176.9 ± 1.3	0.426	0.19
	Weight (kg)	90.8 ± 3.0	94.9 ± 2.6	0.207	0.31
	Percent body fat (%)	19.5 ± 1.1	21.2 ± 1.0	0.302	0.25
	Waist circumference (cm)	93.8 ± 2.1	104.8 ± 5.8	0.083	0.43
	Hip circumference (cm)	105.4 ± 1.5	107.0 ± 1.4	0.427	0.19
	WHR	0.89 ± 0.01	0.98 ± 0.05	0.120	0.39
**Cardiometabolic risk factors**					
	SBP (mmHg)	132.2 ± 2.8	133.3 ± 1.8	0.774	0.07
	DBP (mmHg)	81.0 ± 2.4	83.4 ± 1.8	0.399	0.20
	MBP (mmHg)	101.5 ± 2.3	103.4 ± 1.6	0.499	0.16
	HDL-C (mg/dL)	65.0 ± 4.6	50.9 ± 2.9	0.014	0.64
	TG (mg/dL)	89.9 ± 7.7	159.1 ± 16.2	0.000	1.07
	Fasting glucose (mg/dL)	97.7 ± 1.8	107.1 ± 2.3	0.010	0.74
	TG/HDL	1.5 ± 0.2	3.4 ± 0.5	0.001	0.97
	TyG	8.3 ± 0.1	9.0 ± 0.1	0.000	1.17
**Cognitive function**					
	PVT correctness (score)	38.9 ± 0.3	38.2 ± 0.7	0.470	0.20
	PVT reaction time (msec)	318.8 ± 4.4	325.8 ± 7.7	0.399	0.17
	DMS correct (score)	26.5 ± 0.6	27.3 ± 0.6	0.257	0.28
**Physical fitness**					
	Sit and reach (cm)	25.9 ± 1.9	22.5 ± 2.1	0.214	0.31
	Hand grip strength (kg)	48.1 ± 1.9	48.8 ± 2.0	0.840	0.04
	Vertical Jump (cm)	19.3 ± 1.4	18.4 ± 1.0	0.521	0.16
	Chair sit-stand (*n*)	48.0 ± 2.8	42.8 ± 2.7	0.225	0.32
	VO_2max_ (mL/kg/min)	39.5 ± 3.4	29.3 ± 2.9	0.008	0.80

MetSyn, metabolic syndrome; WHR, waist-to-hip ratio; BP, blood pressure; SBP, systolic BP; DBP, diastolic BP; MBP, mean BP; TG, Triglyceride; TyG, TG/glucose; PVT, psychomotor vigilance task; DMS, delayed-match-to-sample task.

Tables 3, 4 show the results of Spearman’s correlation and the stepwise linear regression analysis between cognitive function and fasting glucose, blood lipid profiles, and surrogate estimates of insulin resistance in US firefighters. HDL-C was significantly correlated with PVT correctness score (R_s_ = 0.37) and DMS reaction time (R_s_ = −0.39). In addition, TG (DMS total time, R_s_ = 0.42; DMS reaction time, R_s_ = 0.46), TG/HDL-C (DMS total time, R_s_ = 0.49; DMS reaction time, R_s_ = 0.42), and TyG (DMS total time, R_s_ = 0.44; DMS reaction time, R_s_ = 0.47) were positively correlated with DMS total time and DMS reaction time. In stepwise linear regression, HDL-C predicted DMS total time (β = −0.44, R^2^ = 0.19) and TyG (β = 0.43, R^2^ = 0.19) predicted DMS reaction time.

**Table 3:** Spearman’s correlations between cognitive function and blood lipid profiles, and surrogate estimates of insulin resistance in US firefighters.

	HDL-C (md/dL)	TG (md/dL)	Glucose (md/dL)	TG/HDL-C	TyG
PVT correctness (score)	0.369^*^	0.033	−0.131	−0.146	0.035
PVT reaction time (msec)	0.096	−0.154	−0.069	−0.143	−0.114
DMS correct (score)	0.009	−0.072	0.090	−0.010	−0.111
DMS total time (msec)	−0.394^*^	0.421^*^	0.209	0.494^**^	0.437^**^
DMS reaction time (msec)	−0.207	0.457^**^	0.271	0.419^*^	0.474^**^

TG, Triglyceride; TyG, TG/glucose; PVT, psychomotor vigilance task; DMS, delayed-match-to-sample task. ^*^p < 0.05, ^**^p < 0.01.

**Table 4:** Stepwise linear regression analysis on cognitive function with blood lipid profiles and surrogate estimates of insulin resistance in US firefighters.

	Model	B	SEM	β	T	Sig.	95% CI	
							Lower	Upper
DMS total time (msec)	(Constant)	9578.494	1445.375		6.627	0.000	6617.777	12539.210
	HDL-C	−61.297	23.640	−0.440	−2.593	0.015	−109.722	−12.873
	R^2^ = 0.194 (*F* = 6.723, *p* < 0.05)							
DMS reaction time (msec)	(Constant)	−7312.768	3993.969		−1.831	0.077	−15481.351	855.815
	TyG	1188.643	461.064	0.432	2.578	0.015	245.662	2131.624
	R^2^ = 0.186 (*F* = 6.646, *p* < 0.05)							

TyG, TG/glucose; DMS, delayed-match-to-sample.

## 4. Discussion

The study aimed to compare differences in cardiometabolic disease risk factors, cognitive function, and physical fitness tests among US firefighters with and without MetSyn. The main finding from the present study shows that US firefighters with MetSyn, compared with non-MetSyn, manifest worse physical and cognitive function, distinguished by (i) higher fasting glucose and TG concentrations, (ii) lower HDL-C concentrations, (iii) higher surrogate estimates of insulin resistance (TG/HDL-C and TyG), (v) slower response in working memory, (iv) and poor CRF. Further, among the firefighters, HDL-C and TyG were associated with DMS total time and DMS reaction time, respectively.

It is widely recognized that MetSyn is positively associated with an increased risk of sudden cardiac death (25). Our study showed that approximately 58% of US firefighters had MetSyn, exceeding more than 1.5 times the US MetSyn prevalence (7). Bour et al. reported that the prevalence of MetSyn was 28.3% for firefighters using the modified Joint Scientific Statement (26), and Carey et al. demonstrated that the prevalence of MetSyn was 46.7% in US firefighters using the National Institute of Health (27). A wide range of MetSyn prevalence trends among US firefighters may be stemmed from various diagnostic criteria and definitions. Furthermore, given that 90% of our study participants are overweight and obese and considering the strong relationship between the degree of adiposity and higher risk for MetSyn, it is expected that we observe a higher prevalence of MetSyn in the present study when compared to others.

The present study demonstrated differences in anthropometric characteristics between US firefighters with and without MetSyn; the US firefighters with MetSyn had significantly higher in all anthropometric characteristics (age, BMI, height, weight, percent body fat, waist, and hip circumference, WHR) compared with the non-MetSyn group. Although the pathophysiology of MetSyn is not clearly understood, it is known that aging and BMI play an important role in the deterioration of metabolic health, i.e., at high risk for MetSyn (28, 29). To exclude the potential confounding effects of different ages and BMI on cardiometabolic disease risk factors, cognitive function, and physical fitness between US firefighters with and without MetSyn, we further performed a rigorous paired-match analysis with respect to age and BMI, confirming that all anthropometric characteristics were not different between the two groups.

Our total cohort and paired-match analysis consistently demonstrated that fasting glucose, blood lipid profiles, and surrogate estimates of insulin resistance were worse in US firefighters with MetSyn vs. without MetSyn. However, the observation that the difference in blood pressure variables among the total cohort disappeared in pair-matched analysis suggests that blood pressure variables are impacted by age and BMI compared with fasting glucose, blood lipid profiles, and surrogate estimates of insulin resistance among US firefighters. Thus, it may be important to match age and BMI while researching firefighters with MetSyn. For the surrogate estimates of insulin resistance, there were large effect sizes determined for MetSyn vs. non-MetSyn among firefighters (Cohen’s *d*; TG/HDL-C = 0.97, TyG = 1.17). This is not surprising since MetSyn is associated with the blood glucose-insulin system. Given that insulin resistance increases cardiovascular event risk in US firefighters (30), firefighters should be given an early clinical diagnosis and regular medical examinations for MetSyn.

Our study revealed that CRF measured by submaximal YMCA cycling test is significantly lower, approximately 35%, in firefighters with MetSyn compared with non-MetSyn, from both cohort and paired-match analyses. Previous studies suggested that higher CRF has been associated with decreased number of metabolic abnormalities among firefighters (31, 32). Especially, McAllister et al. reported that firefighter’s cardiorespiratory fitness (CRF) was negatively associated with surrogate estimated of insulin resistance (HOMA-IR) (33) and lowest CRF levels (Metabolic equivalents; METs ≤ 10) was a higher prevalence of MetSyn compared to highest CRF level (METs > 14) in US firefighter (26). Collectively, increases in CRF might be needed to prevent and treat hypertension, dyslipidemia, dysglycemia, and abdominal obesity among US firefighters.

A previous study suggested that working in an environment of excessive heat in a variety of jobs was associated with an increased risk of cognitive impairments (34). The MetSyn group in the present study showed impaired working memory compared with the non-MetSyn group. In the course of their work, firefighters are exposed to several negative environmental factors, including heat exhaustion and toxic hazards, which can result in negative effects on neuro-cognitive function (35). Canetti et al. investigated the effect of a simulated 15-min firefighting task on psychological and heat stress indicators in firefighters (36). The author confirmed that cognitive function was impaired due to exposure to elevated heat stress and core temperature during simulated firefighter duties. In addition, Hemmatjo et al. examined the effect of simulated firefighting tasks on visual and auditory cognitive function in healthy professional firefighters (37). The author found that auditory cognitive function, working memory, and information processing declined more than visual function following the live-fire task. Firefighting may lead to more central nervous system dysfunction as heat stress induces a negative thermoregulatory response (38). Moreover, thermoregulatory dysfunction deteriorates blood glucose levels, blood vessels, and nerves through sweat glands and dehydration from heat stress. Thus, firefighters with MetSyn are at a higher risk of experiencing impaired cognitive function than those without MetSyn because they have higher resting metabolic rates, heart rate, and body temperatures, making them more susceptible to heat exhaustion (39, 40).

Interestingly, the present study yields valuable information regarding the association between fasting glucose, blood lipid profiles, and surrogate estimates of insulin resistance and cognitive function. Notably, HDL-C was positively associated with sustained attention and negatively associated with working memory response, and TG, TG/HDL, and TyG were significantly correlated with working memory speed and response time. The questions under investigation are whether a firefighter’s fasting glucose, blood lipid profiles, and surrogate estimates of insulin resistance status are marker-specific for cognitive function and whether the risk of decreasing cognitive function is the result of obesity-related cardiometabolic disease risk factors or the result of the firefighter’s hazardous working conditions. In addition, the results of the study suggested that HDL-C and TyG were potential markers of working memory function in US firefighters. He et al. suggested that HDL-C and TG levels were associated with events of mild cognitive impairment (41), and Crichton et al. reported that individuals in the higher HDL-C group (≥60 mg/dL) had a better working memory and mental state than the lower HDL-C group (<40 mg/dL) (42). Although the neuro-physiological mechanism is still unclear, HDL-C eliminates excessively impaired cholesterol and transports it to the liver. This transport pathway could be used to maintain and preserve the specific regions of the frontal and temporal lobes connected with cognitive functions such as attention, problem-solving, inhibition of behavior and memory, etc. (43, 44). Given that cerebral blood flow is associated with impaired memory, attention, and behavior, this could potentially elucidate the relationship between blood lipid profiles and surrogate estimates of insulin resistance and cognitive function. Furthermore, Willmann et al. found that insulin resistance plays an independent role in cognitive function among people with type 2 diabetes (45), and Neergaard et al. reported that long-term follow-up (15 years) of their study revealed that individuals with impaired fasting glucose and insulin resistance had an increased risk of cognitive dysfunction (46). Insulin resistance shares pathophysiological mechanisms with type 2 diabetes and dementia and can be used to estimate memory function through activation in brain glucose metabolism. Therefore, firefighters with MetSyn might lead impaired situational judgments under live-fire conditions.

The strengths of the present study are as follows: (a) first-time simultaneous comparison of cardiometabolic disease risk factors, cognitive function, and physical fitness in US firefighters with MetSyn vs. without MetSyn, (b) examination of the association between MetSyn and cognitive function among US firefighters, (c) a rigorous pair-matching between US firefighters with MetSyn group and non-MetSyn group with respect to age and BMI, (e) estimation of insulin resistance (TG/HDL-C, TyG) to identify risk factor of dysglycemia. Nevertheless, this study has several limitations. One of the limitations of our cross-sectional study was that it examined a small sample size for the total cohort and paired-match analysis, with only one city’s fire department and including male participants only. Future studies should examine larger samples of US firefighters from various geographic locations and include longitudinal follow-up studies. In addition, physical fitness is not a firefighting-specific task test. Therefore, future study is needed to determine whether there is a relationship between firefighter functional fitness test and cognitive function among US firefighters.

## 5. Conclusion

Our study findings demonstrated that US firefighters with MetSyn are predisposed to surrogate markers of insulin resistance and cognitive function, even when matched for age and BMI, and there was a negative association between metabolic disease risk factors and cognitive function in US firefighters. Therefore, the prevention/treatment of MetSyn may be beneficial to supporting firefighters’ safety and occupational performance.

## Data availability statement

The raw data supporting the conclusions of this article will be made available by the authors, without undue reservation.

## Ethics statement

The studies involving human participants were reviewed and approved by #958-2019. The patients/participants provided their written informed consent to participate in this study.

## Author contributions

M-WS, JK, and HJ conceived and designed the experiments. JG and HJ performed the experiments. M-WS, J-ML, KH, JK, and HJ analyzed and interpreted the data. M-WS, JG, J-ML, KH, JK, and HJ contributed reagents, materials, analysis tools or data and wrote the paper. All authors contributed to the article and approved the submitted version.

## Conflict of interest

The authors declare that the research was conducted in the absence of any commercial or financial relationships that could be construed as a potential conflict of interest.

## Publisher’s note

All claims expressed in this article are solely those of the authors and do not necessarily represent those of their affiliated organizations, or those of the publisher, the editors and the reviewers. Any product that may be evaluated in this article, or claim that may be made by its manufacturer, is not guaranteed or endorsed by the publisher.
